# Preschoolers’ anthropomorphizing of robots: Do human-like properties matter?

**DOI:** 10.3389/fpsyg.2022.1102370

**Published:** 2023-02-06

**Authors:** Elizabeth J. Goldman, Anna-Elisabeth Baumann, Diane Poulin-Dubois

**Affiliations:** Department of Psychology, Centre for Research in Human Development, Concordia University, Montréal, QC, Canada

**Keywords:** social robots, Naïve Biology, animacy, interview, preschoolers

## Abstract

Prior work has yielded contradicting evidence regarding the age at which children consistently and correctly categorize things as living or non-living. The present study tested children’s animacy judgments about robots with a Naïve Biology task. In the Naïve Biology task, 3- and 5-year-olds were asked if robots, animals, or artifacts possessed mechanical or biological internal parts. To gauge how much children anthropomorphize robots in comparison to animals and artifacts, children also responded to a set of interview questions. To examine the role of morphology, two robots were used: a humanoid robot (Nao) and a non-humanoid robot (Dash). To investigate the role of dynamic characteristics, children saw one robot behave in a goal-directed manner (i.e., moving towards a ball) and one robot exhibit non-goal-directed behavior (i.e., moving away from a ball). Children of both age groups correctly attributed biological insides to the animal and mechanical insides to the artifact. However, 3-year-olds seemed confused about what belonged inside both robots and assigned biological and mechanical insides equally. In contrast, 5-year-olds correctly assigned mechanical insides to both robots, regardless of the robot’s morphology or goal-directedness. Regarding the Animacy Interview, 3-year-olds performed at chance level when asked about the animacy of robots, animals, and artifacts. In contrast, 5-year-olds correctly attributed animacy to animals and accurately refrained from anthropomorphizing artifacts and the non-humanoid robot Dash. However, 5-year-olds performed at chance for Nao, suggesting they may be confused about the psychological properties of a human-looking robot. Taken together, these findings reveal a developmental transition during the preschool years in the attribution of biological and psychological properties to social robot.

## Introduction

Young children’s judgments of the animacy of both living and non-living things has been a topic of interest in developmental science for decades (Piaget et al., 1929; Ochiai, 1989; Stavy and Wax, 1989; Hatano et al., 1993). Animacy cues include properties like autonomous movement, gestures, goal-directedness, gaze following, contingent behaviors, speech, and emotions (Itakura et al., 2008; O’Connell et al., 2009; Meltzoff et al., 2010; Beran et al., 2011; Somanader et al., 2011; Breazeal et al., 2016; Kennedy et al., 2017; Manzi et al., 2020). Though related, animacy and aliveness (i.e., is something living or non-living) are separate constructs. Something can be living, but exhibit lower levels of animacy (e.g., plants), and something can be non-living but have higher levels of animacy (e.g., robots). However, researchers have found contradicting evidence regarding the age at which children consistently and correctly categorize things as living or non-living. Some researchers believe this ability emerges in early childhood, as early as 4 or 5 years of age (Piaget et al., 1929; Pérez Rodríguez, 1985; Inagaki and Hatano, 1996; Wright et al., 2015). For example, Inagaki and Hatano (1996) interviewed children and asked them whether different things (i.e., plants, animals, and artifacts) were living. The results indicated that the majority of five-year-olds thought both plants and animals were living things. However, other researchers have reported that children are not capable of correctly categorizing all living things until they are 9 or 10 years of age (Carey, 1985; Richards, 1989; Venville, 2004; Leddon et al., 2008). In one study, Leddon et al. (2008) tested 4- to 10-year-olds in a short categorization game at their schools and found that children at 9 and 10 years old still failed to reliably and consistently classify plants as living things.

To address this controversy, researchers have examined what type of information children rely on in making their judgments about animacy. Prior work suggests that children often use both static and dynamic characteristics (e.g., autonomous movement, facial features, gaze following) to determine whether something is alive or not (Richards and Siegler, 1986; Ochiai, 1989; Richards, 1989; Stavy and Wax, 1989; Rakison and Poulin-Dubois, 2001; Opfer and Siegler, 2004; Waxman and Medin, 2006). A body of literature has emerged that assesses children’s

conceptual understanding of living and non-living things (Gottfried and Gelman, 2005; Jipson and Gelman, 2007; Wright et al., 2015). Like human social partners, social robots display a range of animacy characteristics in their social interactions and conversations (Arita et al., 2005; Itakura et al., 2008; O’Connell et al., 2009; Meltzoff et al., 2010; Beran et al., 2011; Somanader et al., 2011; Breazeal et al., 2016; Ahmad et al., 2017; Kennedy et al., 2017; Manzi et al., 2020).

One way to measure animacy is to examine how much children attribute human characteristics or behaviors (i.e., anthropomorphize) to different animals, artifacts, or robots. For example, attributing emotions like anger or happiness to an inanimate object or a robot would be anthropomorphizing. Which characteristics are the most critical to trigger anthropomorphic judgments about robots in young children remains an open question. In 2015, Wright and colleagues presented 4- and 5-year-olds with static images of humans, animals, and vehicles. The five-year-olds correctly categorized humans and animals as animate and vehicles as inanimate. Crucially, five-year-olds were above chance but failed to perform at the levels adults do (Wright et al., 2015). In the present work, we focus on a special category of artifacts (robots) whose movement and appearance can be manipulated to identify the object properties that trigger anthropomorphism. Specifically, robots though mechanical in nature can be designed and programmed to exhibit a variety of animacy characteristics (e.g., human appearance, autonomous movement, gestures, goal-directedness, contingent behaviors, speech, and emotions). In work that examined adults, Broadbent et al. (2013) found that adults preferred a robot with a human-like face compared to a robot that had a silver face or no face. Importantly adults rated the robot with the human-like face as having more personality and less eeriness.

In a recent article, Clark and Fischer (2022) concluded that “the more social cues robots display, the more competent they are judged to be by adults” (p. 19). Existing literature suggests that children react to robots in a similar way. For example, infants are more likely to follow a robot’s gaze if the robot acts in a socially contingent manner (Itakura et al., 2008; Meltzoff et al., 2010; Peca et al., 2016) and young children show a preference for a robot that displays contingent behavior over a non-contingent robot (Breazeal et al., 2016). There are many characteristics that both human and non-human (e.g., robotic) social partners display during their interactions and conversations with others. Of interest to the present study are goal-directed behavior and autonomous movement.

Prior research has demonstrated that young children will correctly judge plants and animals as living when they detect goal-directed motion (Opfer and Siegler, 2004). A similar pattern has also been found with inanimate agents. For example, infants as young as 3-months attribute goal-directed actions to a self-propelled box (Luo, 2011). Infants recognize goal-directed actions in robots as well. In another study, infants (24-to 35 months old) imitated a robot’s actions (i.e., put beads into a cup) if the robot made eye contact (Itakura et al., 2008). In addition to goal-directed actions, young children often justify agents (e.g., animals and people) as alive because they can move autonomously (Richards and Siegler, 1986; Venville, 2004; Tao, 2016; Fouquet et al., 2017).

Infants and young children have been found to readily differentiate between an autonomous and a non-autonomous moving robot. In one of the first studies to expose children to a self-propelled robot, infants as young as 9 months considered anomalous such objects as reflected in negative affect and increased attention (Poulin-Dubois et al., 1996). Other research revealed that infants expect an autonomous robot to be spoken to in a social interaction (Arita et al., 2005; Somanader et al., 2011; Chernyak and Gary, 2016). For instance, Chernyak and Gary (2016) found that children who saw an autonomous robot were more likely to say the robot could feel upset or experience other emotions than children who saw the non-autonomous robot. Thus, in the present study, the robots demonstrated the animacy characteristics of goal-directedness and autonomous movement to examine whether such characteristics, in combination with the robot’s morphology, would impact how animate children judge these robots to be.

An important question is how morphology can influence children’s perceptions of robots. Much of the existing work on children’s perception of robots has asked them to conceptualize a single robot (Breazeal et al., 2016; Chernyak and Gary, 2016; Peca et al., 2016; Brink and Wellman, 2020; Manzi et al., 2020). Importantly, robots can vary significantly in their physical appearance or morphology. Fong et al. (2003) concluded that the morphology of a robot influences how others will ultimately interact with it. For example, a robot that is designed to resemble a dog will be treated differently than a robot that is more humanoid in appearance. Fong et al. (2003) go on to say that morphology can alter the capabilities (e.g., what actions the robot can do or perform) of social robots. In a recent review paper, van Straten et al. (2019) report that the findings in the field are mixed regarding whether robot features, like morphology, predict children’s trust in robots. Crucially, younger children tended to pay more attention to the robot’s morphology, particularly its anthropomorphism, than older children (van Straten et al., 2019). In another review paper, Marchetti et al. (2018) show that interaction with non-human social agents is preferred when facial features and gaze following mirror those of human agents. Kamewari et al. (2005) demonstrated that 6.5-month-olds have the ability to attribute agency to both human and robot emotions but are unable to do so when the robot lacks a human-like appearance. Thus, morphology was manipulated directly in the present study by using two robots that vary in appearance: a humanoid (Nao) and a non-humanoid (Dash) robot.

Previous work has examined how a robot’s human appearance impacted whether children shared with a robot (Nijssen et al., 2021). In one study, 4- to 9-year-olds were shown videos of a humanoid robot (Nao) and a non-humanoid robot (LEGO Mindstorm). One of the robots demonstrated having feelings (i.e., by contingently answering the question “What makes you happy?”), while the other robot did not demonstrate the ability to have feelings (i.e., answered the question “What makes you happy?” by responding, “I do not know because I cannot feel happy or sad”). Although older children shared more with the robots than younger children, children in both age groups shared more with the robot that demonstrated emotion, regardless of the robot’s appearance. Thus, in this case, the ability to demonstrate emotion trumped the robot’s morphology.

The present study builds upon prior work by investigating how the morphology (human or non-human appearance) of social robots may impact children’s perceptions of them. We examined anthropomorphic judgments by contrasting robots that differed in their physical appearance. Through this, we can better examine what role, if any, morphology plays in young children’s conceptualization of robots. Specifically, we aimed to determine if the appearance of the robot impacts whether children attribute biological and psychological properties to the robot.

As discussed above, prior work has shown that infants and young children behave similarly in their interactions with both robots and people (O’Connell et al., 2009; Meltzoff et al., 2010; Okumura et al., 2013; Mwangi et al., 2018). However, evidence that children can learn from and interact with robots is not sufficient to conclude whether children view robots as social agents. To better understand this, we must examine how children conceptualize and categorize robots. Several tasks have been designed to answer this question, including the use of interviews.

Interviews are a widely used measure in the body of literature to examine children’s abilities to differentiate living from non-living things (Carey, 1985; Stavy and Wax, 1989; Hatano et al., 1993; Tao, 2016; Fouquet et al., 2017; Kim et al., 2019). Additionally, interviews can be an effective measure for assessing children’s perceptions of robots, as interview questions can cover a variety of domains (Jipson and Gelman, 2007; Beran et al., 2011; Chernyak and Gary, 2016; Manzi et al., 2020; Goldman, 2022). The interview questions children are asked about social robots tend to address the following domains: Mental (e.g., Can the robot think?), perceptual (e.g., Can the robot see?), social (e.g., Could you trust the robot with a secret?), emotional (e.g., Does the robot have feelings?) and biological (e.g., Is the robot alive?). For example, Okanda et al. (2021) examined whether 3- and 5-year-olds’ perceptions of the Kirobo robot had changed after a social interaction. The Kirobo robot stood upright and had a torso, legs, arms, and a head with eyes and a mouth. Children were asked a series of interview questions (e.g., Does this one eat?, Can this one break?, Does this one think?, Does this one see things?) and were then allowed to interact with and talk to the robot. After the interaction, the children were asked the same interview questions again. The results showed that the 3-year-olds were more likely to attribute biological properties to the robot than the 5-year-olds. However, the 3-year-olds attributed fewer biological properties to the robot after the social interaction. Similarly, in a study by Kim et al. (2019), 3-, 4-, and 5-year-olds were asked whether a humanoid robot (Vex, produced by Vex Robotics) was alive. The younger children in the study were more likely to say the humanoid robot was alive than the older children.

In a study by Okita and Schwartz (2006), children 3 through 5 years of age interacted with either a high or low-contingency robot. Children were randomly assigned to interact with one of four different robotic animals (NeCoRo Cat, BN-1 Cat, FurReal Cat, or Paro Seal Robot). During the interaction, the robots generated sound and moved. However, only the high-contingency robots made sounds or moved in reaction to what the child did. During the interaction, children answered questions about the robot’s biology (e.g., needs to have food?, Can hear?, Is alive?). Older children were less likely than younger children to attribute biological properties to the robots. Regarding the contingency of the robot, older children attributed fewer biological properties to the low contingent robot, but the contingency of the robot had no effect on the 3-year-old’s attributions of biological properties. Younger children were more likely to say the low contingent robot was alive, whereas older children were more likely to say the high contingent robot was alive.

As interviews require verbal responses, they are not a suitable measure for infants and younger children. Other methods that do not rely solely on verbal responses can be used to assess whether children depict robots as social agents. For example, Gottfried and Gelman (2005) used a Naïve Biology task to examine whether young children attributed biological or mechanical insides to unfamiliar animals, artifacts, and plants. The 3-, 4-, and 5-year-olds were asked to select whether something biological (e.g., lungs) or mechanical (e.g., wires) belonged inside the animal, artifact, or plant. Overall, the 3-year-olds attributed significantly fewer accurate internal parts than the 4- and 5-year-olds. These results suggest that with age, children become better at judging whether animals, artifacts, and plants are more biological or mechanical internally. One might ask whether children would attribute mechanical or biological insides to something ambiguous (e.g., has animate and inanimate characteristics), like social robots.

The present study examined how children conceptualize robots in comparison to novel animals and artifacts. Using a modified version of Gottfried and Gelman’s (2005) Naïve Biology task, children were asked to determine whether something biological (e.g., Heart) or mechanical (e.g., Gears) belonged inside the robots, animal, or artifact. To gauge how much animacy children attributed to the animals, artifacts, and robots, children responded to a set of interview questions that assessed both the psychological (e.g., The ability to think, talk, and feel) and the biological characteristics (e.g., Is it alive?) of the agents. To examine how the morphology of the robot impacted children’s perception and categorization, we used two different robots that varied in their appearance. One robot (Nao) was humanoid in appearance, while the other (Dash) was a non-humanoid-looking robot.

Since young children tend to be more biased by social cues in their interactions with robots, we predicted that the 3-year-olds would be more likely to attribute biological insides to the humanoid robot compared to the non-humanoid robot. We hypothesized that the older children, the 5-year-olds, would attribute mechanical insides to both robots, as children this age have more experience with mechanical artifacts. Secondly, we predicted that children, regardless of age, would judge the humanoid robot (Nao) to be more animate than the non-humanoid robot (Dash). Specifically, we expected interviews compared to the more applied Naïve Biology task to be more challenging for children as interviews are more theoretical and hypothetical in nature. As social robots tend to exhibit dynamic characteristics, we opted to vary the goal-directedness of the robots. Children saw one robot behave in a goal-directed manner (i.e., moved towards a ball), and one robot exhibited non-goal-directed behavior (i.e., moved away from a ball). Which robot behaved in a goal-directed manner was counterbalanced. Thirdly, we expected 3- and 5-year-olds to attribute more animacy to a goal-directed robot, regardless of the robot’s morphology.

## Method

### Participants

Participants included 44 3-year-olds (females = 24; *M_age_* = 42 months, 13 days; range = 36 months, 4 days to 48 months, 0 days) and 45 five-year-olds (females = 26; *M_age_* = 65 months and 2 days; range = 60 months, 2 days to 71 months, 25 days). An *a priori* G*Power 3.1 analysis (Faul et al., 2007) was run to determine the appropriate sample size for a 2 × 4 repeated measures analysis of variance (ANOVA). Our goal was to obtain 0.80 power to detect a moderate effect size of .25 at the standard 0.05 alpha error probability. The analysis revealed a minimum sample size of 40 per group. Therefore, our sample sizes of 44 and 45 children slightly exceed the minimal power requirements. Most children were Caucasian (*n* = 38) and Asian (*n* = 16), with the remainder of the sample identifying as African, Hispanic, or mixed race (*n* = 35). Parents reported on their child’s exposure to robots. In the sample, nine families reported having a robot at home (10.1%), five parents reported that their child interacts with a robot on a regular basis (6.0%), and 18 parents reported that their child regularly played a video game and/or watched a television show that featured robots (20.2%). Many of the families who participated in the study reported high family income (more than $150,000 = 43.1%, $100,000 to $150,000 = 26.9%). Regarding parental education, 66.2% of parents in our sample reported having an advanced degree, 26.9% of parents had completed their bachelor’s degree, and the remainder of parents had completed trade school, high school, or some college.

Participants were recruited from an existing University database of participants. Half of the families were recruited from various states in the United States (the states where most of our participants resided were California, Illinois, and New York; *n* = 46), and the other half from Québec, Canada (*n* = 43). Children from Canada were tested in their preferred language, either English (*n* = 30) or French (*n* = 13). All children from the United States were tested in English (*n* = 46). Six additional participants were tested but excluded for having one of the robots featured in the study or a robot that looked similar at home (*n* = 3), for parental interference (*n* = 1), or for being distracted/disengaged from the task (*n* = 2). Parents received a gift card as compensation, and children received a certificate of merit for their participation. Ethical approval to conduct the current study was granted by the university’s Human Research Ethics Committee (#10000548).

### Materials

**Figure 1:**
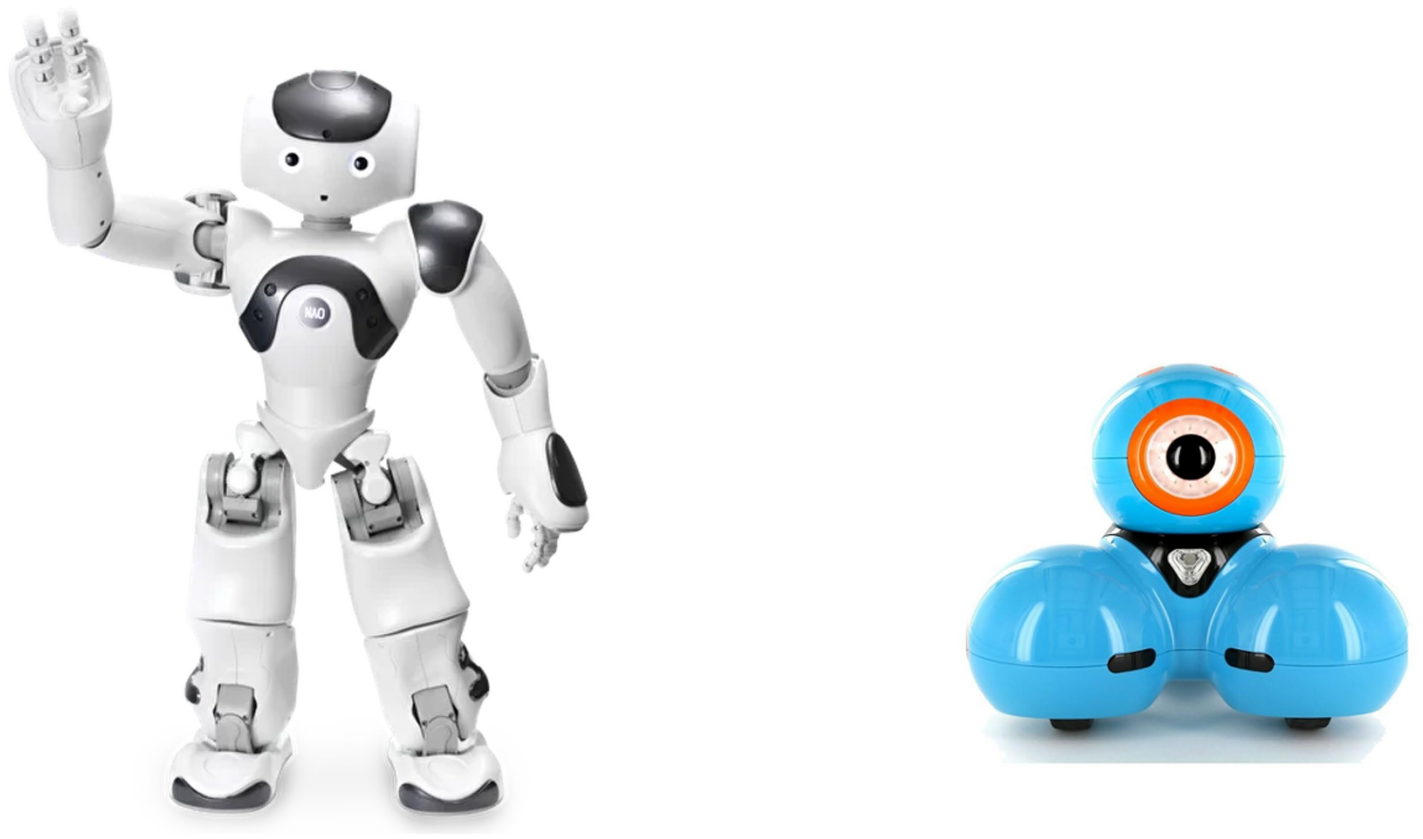
The humanoid robot Nao (left) and the non-humanoid robot Dash. The humanoid robot Nao (left) [reproduced with the permission of Aldebaran; NAO (c) Aldebaran, part of United Robotics Group] and the non-humanoid robot Dash (right) [reproduced with the permission of Wonder Workshop].

Two robots, Nao (a humanoid robot) and Dash (a non-humanoid robot) were used in the present work. The Nao robot (SoftBank Robotics) stood upright, had a torso, arms, legs, and a head with eyes and a mouth. In contrast, the Dash Robot (Wonder Workshop) was light blue in color, rolls around, has a round body, a head, and a single eye (see Figure 1 for pictures of both robots). Prior to selecting the robots for the study, a short survey was administered to undergraduate students (*n* = 23) enrolled in courses at a university. Survey participants were shown pictures of five different robots (Cozmo, Dash, DRK 8080, Nao, and Robie Sr.) and asked to rate how human-like each robot looked. Participants responded on a 5-point Likert scale (1 = not human looking at all to 5 = very human-like). Given that one of our main goals was to examine how morphology impacted young children’s perceptions of robots, we selected the robot that was rated least human-like (Dash, *M* = 1.652, *SD* = 1.005) and the robot that was rated most human-like (Nao, *M* = 4.087, *SD* = 0.928). According to the survey ratings, Nao was rated as significantly more human-looking than Dash [*t*(22) = −13.04, *p* < 0.001, *d* = −2.72]. Other materials included a set of pictures depicting four animals, four artifacts, four mechanical parts, and four biological parts. These pictures were featured in the tasks and were identical to the original stimuli used by Gottfried and Gelman (2005).

### Procedure and design

Parents and children participated online *via* the Zoom video conferencing platform. The Zoom session took approximately 10–15 minutes to complete. Prior to participation, parents completed a consent form and a demographics questionnaire. The Zoom appointment began with a short warm-up session during which the experimenter introduced themselves to the child and attempted to build rapport with the child. The experimenter briefly went over the study procedure, asked the parent to provide verbal consent for participation, and provided a few reminders (i.e., parents should let their child answer independently and refrain from labeling the pictures on the screen).

The study was a between-subjects design and consisted of two conditions, randomly assigned. Each child saw four items: two robots (Nao and Dash), an animal, and an artifact. The only difference between the two conditions was which robot exhibited goal-directed behavior (in condition 1, Dash was goal-directed; in condition 2, Nao was goal-directed). Each condition consisted of two tasks: (1) the Naïve Biology task and (2) the Animacy Interview. Both the order of the four items (artifact, animal, Nao robot, and Dash robot) and the order of the tasks (Naïve Biology and Animacy Interview) were counterbalanced. The two trials that featured robots (Nao and Dash) began with a short video. Each video was approximately 8 s in length. In the video, the robot would either show goal-directed behavior (i.e., moved towards a ball) or non-goal-directed behavior (i.e., moved away from the ball). The robot that behaved in a goal-directed manner (Nao or Dash) and the location of the ball (i.e., on the right or left on the screen) were also counterbalanced across participants. Before starting the video, the experimenter said, “Watch the robot.” After the video had finished, the experimenter proceeded with either the Naïve Biology or the Animacy Interview task.

#### Naïve Biology task

The Naïve Biology task was adapted from Gottfried and Gelman (2005). The present study featured four item types: two sets of unfamiliar items (animals, artifacts) and two different unfamiliar robots (Dash and Nao). Although each participant was administered only one artifact and one animal trial, there were four different animals (i.e., ibek, pacarana, tapir, and cavy) and four different artifacts (i.e., intercom, espresso maker, voice recorder, and electric razor) used across participants. Which child saw which animal or artifact was counterbalanced. These four animals and four artifacts, as well as the pairs of internal parts (described below in more detail), were identical to those used in the original Naïve Biology task pioneered by Gottfried and Gelman (2005), except that two robots were added for the present study.

**Figure 2:**
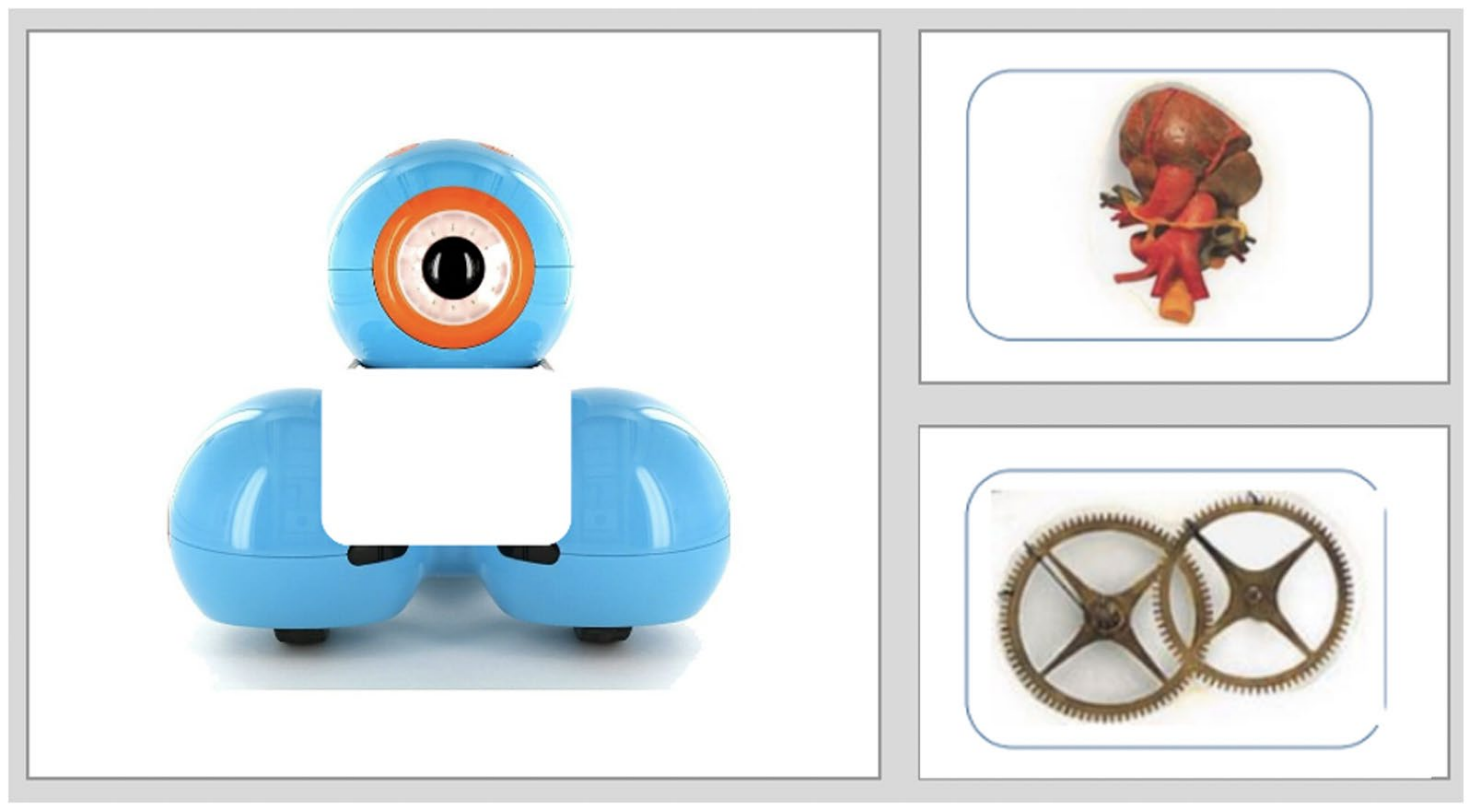
A Dash robot trial from the Naive Biology task. Children selected a biological (i.e., top in this example) or mechanical inside (i.e., bottom in this example) for the robot. Dash image (left) reproduced with the permission of Wonder Workshop.

**Figure 3:**
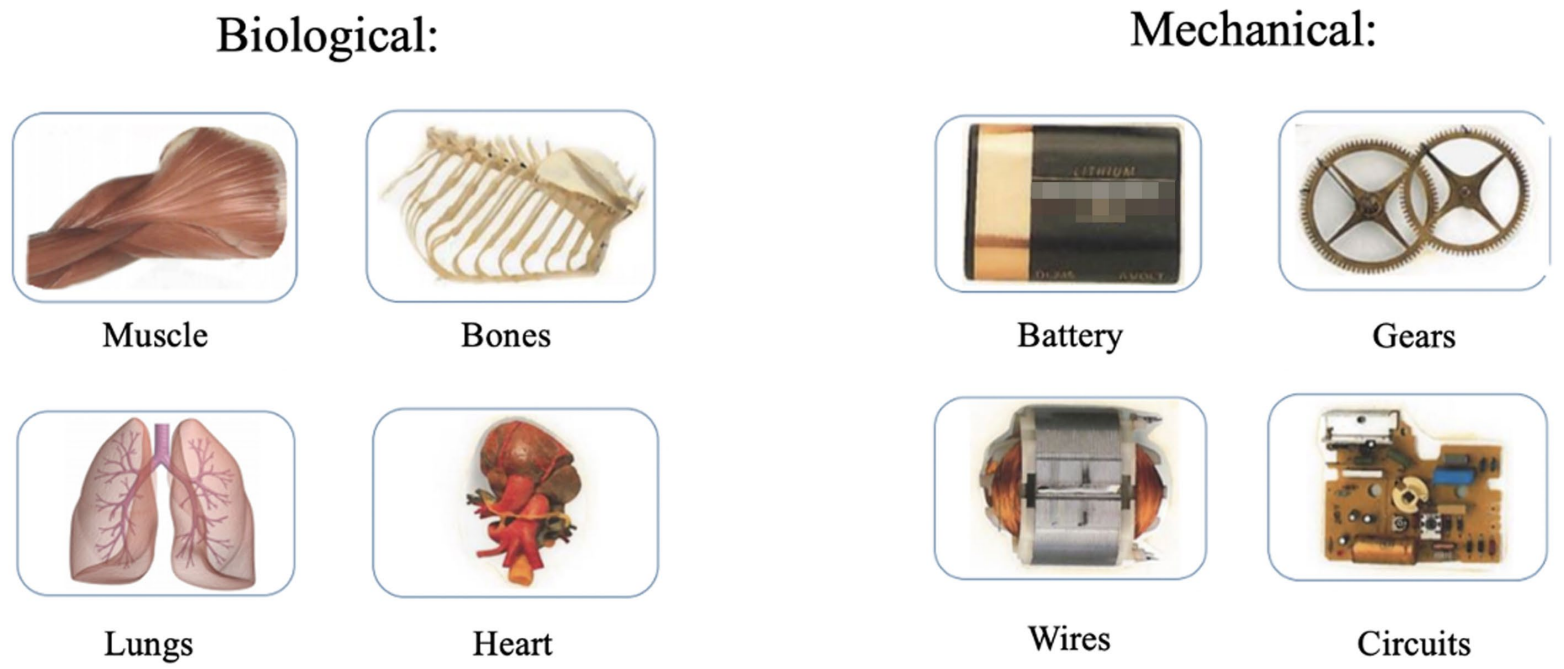
Stimuli (internal parts) for the Naïve Biology task. Biological and mechanical parts were randomly paired together for children to select on each trial of the Naïve Biology task.

The unfamiliar items (i.e., the animal, artifact, Dash, or Nao) were pictured on the screen’s left side, with a white box in the center of the image indicating the “missing part.” The two internal parts were pictured on the right side of the screen (see Figure 2). One of the internal parts was mechanical (i.e., gears, circuits, batteries, wires), and the other was biological (i.e., muscle, lungs, heart, bones). The mechanical and biological internal parts are shown in Figure 3. The placement of the items (mechanical and biological) was counterbalanced so that the biological item was sometimes pictured on top and sometimes pictured on the bottom. In the first trial, the experimenter began the task by saying, “Look, there is a missing part. I need your help. What goes inside?” The experimenter pointed to the white square (i.e., the missing part). Next, the experimenter said, “What goes inside? The top one or the bottom one?” Again, the experimenter pointed to each of the internal parts as they said top and bottom. Children provided their answers verbally (i.e., by saying either top or bottom). If children were too shy to respond verbally, the experimenter prompted the child to point to their selection and asked the parent to confirm whether the child had pointed to the top or bottom internal part. After the child had made their selection, the experimenter moved the indicated internal part (i.e., the top or bottom) into the white square (“the missing part”). The experimenter then said, “Like this?” and waited for the child to confirm they had moved the right part inside. If the child indicated their desired internal part had been moved inside, the experimenter moved on to the subsequent trial. If the child indicated the experimenter had moved the incorrect part inside, the experimenter moved the internal part back to its original starting position and re-prompted the child by asking whether the top or bottom went inside.

The Naïve Biology task consisted of 16 total trials, four per item. Each time the image of the unfamiliar item was paired with different mechanical and biological internal parts. Both the pairing of the internal parts with the items and the order in which the items were presented were counterbalanced. The experimenter refrained from labeling any of the internal parts or items. Additionally, the experimenter never confirmed that the child made a “correct” selection (i.e., the mechanical part inside the artifact or the biological part inside the animal) and refrained from praising the child after they had selected their choice of internal part.

#### Animacy Interview task

For each item (artifact, animal, Nao, Dash), the child was asked a series of interview questions. The Animacy Interview consisted of four questions per item for a total of 16 questions. The questions were: (1) Can the robot/animal/artifact talk? (2) Does the robot/animal/artifact have feelings, like you do? (3) Can the robot/animal/artifact think? and (4) Is the robot/animal/artifact alive? Children were asked to verbally respond “yes” or “no” to each question. If children failed to provide a verbal answer but nodded “yes” or “no,” it was also accepted as answers to the interview questions. After the child provided their yes or no answer, the experimenter would repeat their answer before moving on to the next question. The interview questions were always asked in the same order. During the Animacy Interview, a picture (without any missing part) of the unfamiliar item (artifact, animal, Nao, Dash) was displayed.

## Results

### Coding and scoring

For the Naïve Biology task, children received a point each time they associated a mechanical inside to the four items (animal, artifact, Nao, and Dash). As there were four Naïve Biology trials for each item, the scores per item ranged from 0 to 4. Therefore, higher scores on all trials, except animal, represent better performance. For the Animacy Interview, children received a point each time they answered “yes” for all four items. Children were asked four questions per item: three psychological (think, feel, and talk) and one biological (alive). Therefore, higher scores indicate higher anthropomorphism to the items.

The main analyses (overall ANOVAs for Naïve Biology and Interview) were checked for gender (male or female) and testing language (French or English) covariate effects. No significant interactions were found between gender, testing language, and either the Naïve Biology task or the Interview. Therefore, gender and language were collapsed across all analyses and are not reported below. If a deviation from normality was found, appropriate corrections were applied. If these corrections changed significance, it is discussed below.

First, we examined children’s performance on the two different tasks. Each age group was analyzed independently to determine if the age groups performed differently on each task. Then, the two ages were combined for each task and analyzed. Finally, goal-directedness and robot type were also investigated as possible predictors.

#### Naïve Biology task

**Figure 4:**
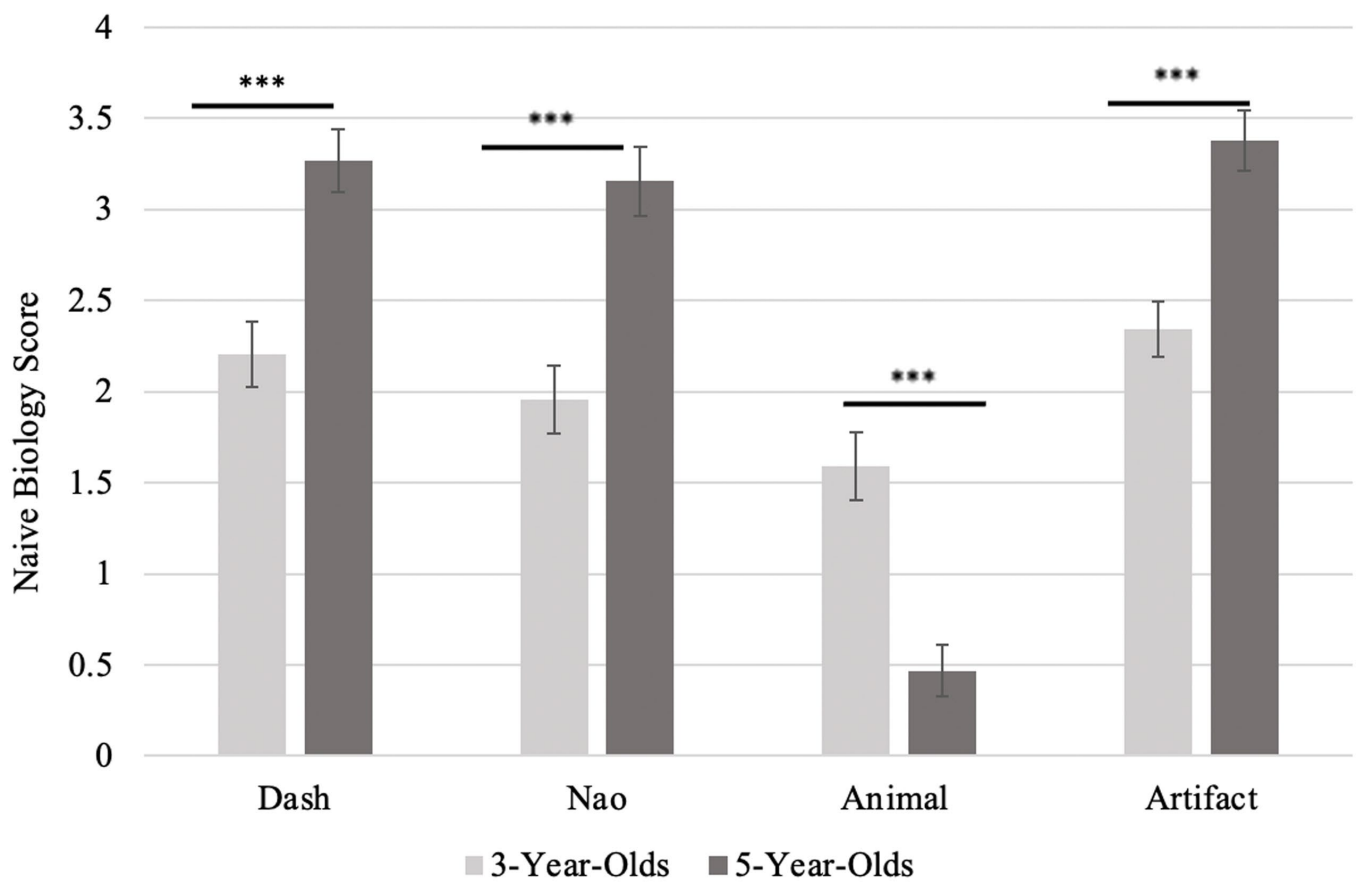
Score on Animacy Interview by item type, as a function of age. Error bars represent standard error. Higher scores represent more anthropomorphizing (with a maximum score of 4). The *** (*p* <0.001) indicates a significant difference between the two age group on that item.

We first examined whether children performed at, above, or below chance for each item (see Table 1). The 3-year-old children performed at chance, as indicated by one-sample *t*-tests, on the Naïve Biology task for both Dash (*M* = 2.21, *SD* = 1.19, *t*(43) = 1.14, *p* = 0.26, *d* = 0.17, 95% CI [−0.13, 0.47]) and Nao (*M* = 1.96, *SD* = 1.24, *t*(43) = −0.24, *p* = 0.81, *d* = −0.04, 95% CI [−0.33, 0.26]). Thus, on average, 3-year-olds equally associated a biological and mechanical inside to both robots featured in the study. A majority of the 3-year-olds (75%) associated the robots with a mechanical inside only part of the time (1 to 3 times out of 4 trials), demonstrating that they were unsure about a robot’s insides (see Table 2). However, the 3-year-old children performed well, scoring above chance on both the artifact trials (*M* = 2.34, *SD* = 1.01, *t*(43) = 2.24, *p = 0*.03, *d* = 0.34, 95% CI [0.03, 0.64]) and the animal trials (*M* = 1.59, *SD* = 1.25, *t*(43) = −2.18, *p = 0*.04, *d* = −0.33, 95% CI [−0.63, −0.02]; see Figure 4).

**Table 1:** Mean scores and chance analyses for the Animacy Interview and Naïve Biology task, as a function of age.

Task	Age	Dash	Nao	Animal	Artifact
Naïve biology	3-year-olds	*M* = 2.21, *SD* = 1.19, *t*(43) = 1.14, *p* = 0.26, *d* = 0.17	*M* = 1.96, *SD* = 1.24, *t*(43) = −0.24, *p* = 0.81, *d* = −0.04	*M* = 1.59, *SD* = 1.25, *t*(43) = −2.18, *p = 0*.04, *d* = −0.33*	*M* = 2.34, *SD* = 1.01, *t*(43) = 2.24, *p = 0*.03, *d* = 0.34*
	5-year-olds	*M* = 3.27, *SD* = 1.18, *t*(44) = 7.23, *p < 0.*001, *d* = 1.08***	*M* = 3.16, *SD* = 1.28, *t*(44) = 6.06, *p < 0*.001, *d* = 0.90***	*M* = 0.47, *SD* = 0.94, *t*(44) = −10.90, *p < 0*.001, *d* = −1.63***	*M* = 3.38, *SD* = 1.09, *t*(44) = 8.46, *p <* 0.001, *d* = 1.26***
Animacy interview	3-year-olds	*M* = 2.18, *SD* = 1.45, *t*(43) = 0.83, *p* = 0.41, *d* = 0.13	*M* = 2.21, *SD* = 1.52, *t*(43) = 0.89, *p* = 0.38, *d* = 0.14	*M* = 2.18, *SD* = 1.32, *t*(43) = 0.92, *p* = 0.37, *d* = 0.14	*M* = 1.71, *SD* = 1.59, *t*(43) = −1.23, *p* = 0.23, *d* = −0.19
	5-year-olds	*M* = 1.42, *SD* = 1.45, *t*(44) = −2.67, *p* = 0.01, *d* = −0.40*	*M* = 1.93, *SD* = 1.50, *t*(44) = −0.30, *p* = 0.77, *d* = −0.04	*M* = 2.91, *SD* = 0.82, *t*(44) = 7.45, *p* < 0.001, *d* = 1.11***	*M* = 0.47, *SD* = 0.73, *t*(44) = −14.17, *p* < 0.001, *d* = −2.11***

*** (*p* < 0.001) indicate significance for that chance analysis.

* (*p* < 0.05) indicate significance for that chance analysis.

**Table 2:** Distribution of mechanical responses for Dash and Nao.

Number of mechanical responses	3-year-olds		5-year-olds	
	Dash	Nao	Dash	Nao
0	4	7	2	4
1	8	9	3	1
2	14	11	5	6
3	11	13	6	7
4	7	4	29	27

The “Number of Mechanical Responses” refers to the number of trials (0–4) a participant chose the Mechanical inside for the robots (Nao or Dash). The count represents the number of children who chose the mechanical inside over the four trials.

In comparison, 5-year-old children performed above chance on all four items, Dash (*M* = 3.27, *SD* = 1.18, *t*(44) = 7.23, *p < *0.001, *d* = 1.08, 95% CI [0.71, 1.44]), Nao (*M* = 3.16, *SD* = 1.28, *t*(44) = 6.06, *p < *0.001, *d* = 0.90, 95% CI [0.55, 1.25]), animal (*M* = 0.47, *SD* = 0.94, *t*(44) = −10.90, *p < *0.001, *d* = −1.63, 95% CI [−2.07, −1.17]), and artifact (*M* = 3.38, *SD* = 1.09, *t*(44) = 8.46, *p <* 0.001, *d* = 1.26, 95% CI [0.86, 1.65]). As seen in Table 2, the majority of the older children correctly categorized both robots as mechanical most of the time. They also correctly categorized artifacts as mechanical and animals as biological. There is thus a developmental progression in children’s’ understanding of robots’ animacy.

**Figure 5:**
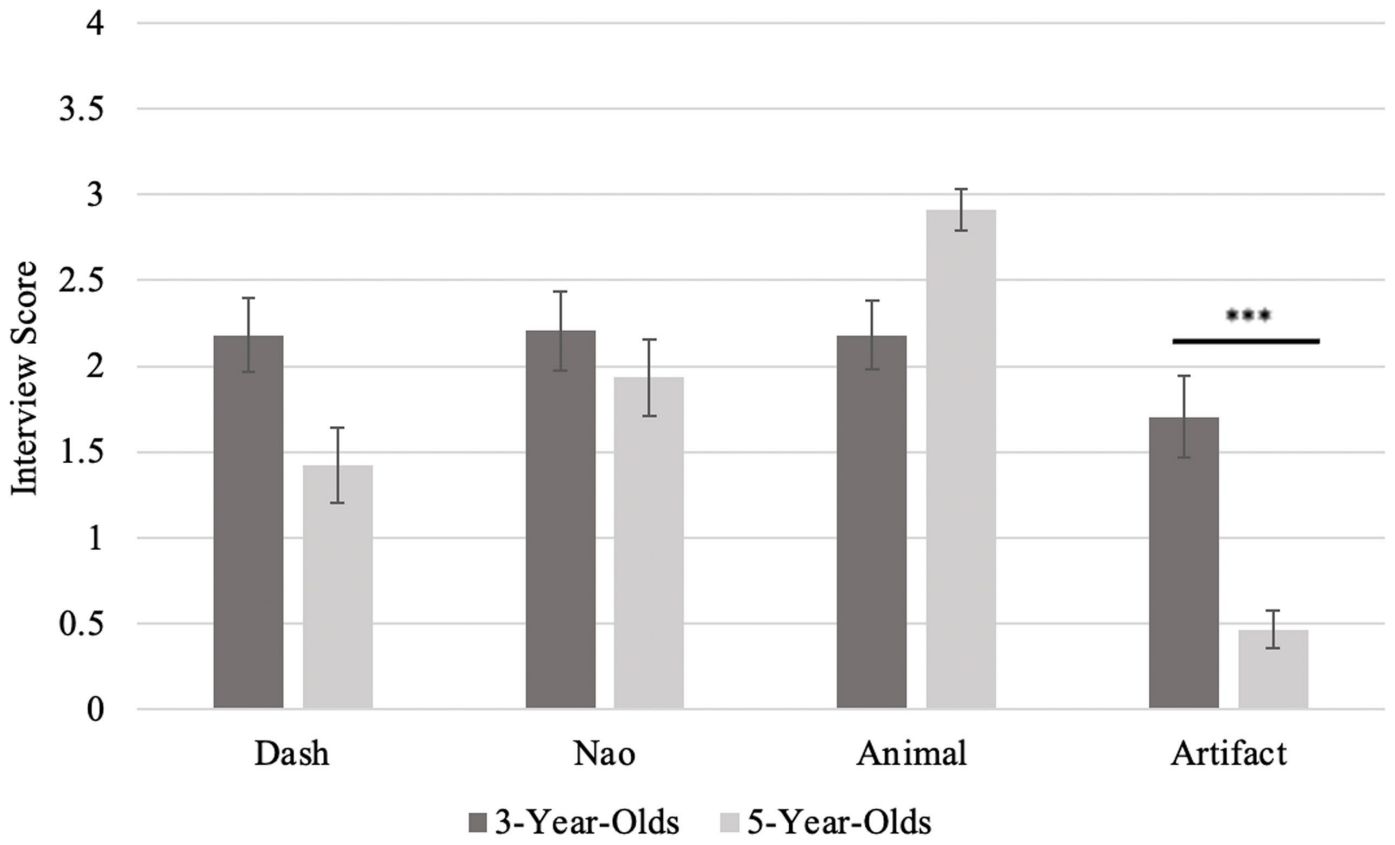
Score on animacy interview by item type, as a function of age. Error bars represent standard error. Higher scores represent more anthropomorphizing (with a maximum score of 4). The *** (*p* < 0.001) indicates a significant difference between the two age group on that item.

A repeated-measures analysis of variance test (ANOVA) was conducted to compare Naïve Biology task performance across the different items (Nao, Dash, animal, and artifact), with age as a between-subjects factor. A main effect of both items [*F*(3) = 59.64, *p* < 0.001, ηp^2^ = 0.41] and age [*F*(1) = 12.83, *p* < 0.001, ηp^2^ = 0.13] was found. As expected, the interaction between items and age was also significant [*F*(3) = 25.44, *p* < 0.001, ηp^2^ = 0.23]. *Post hoc* analyses revealed that naïve biology performance differed between the animal and artifact trials [*t*(88) = −1.83, *p_holm_* < 0.001], as well as between the Dash and the animal trials [*t*(88) = 1.71, *p_holm_* < 0.001], and the Nao and animal trials [*t*(88) = 1.53, *p_holm_* < 0.001]. Thus, children correctly believed that artifacts are more mechanical (or less biological) than either the animals or the robots (Nao and Dash). The *post hoc t*-tests revealed that the 5-year-olds significantly outperformed the 3-year-olds on all items [*t*(88) > −4.92, *p_holm_* < 0.001], see Figure 5.

#### Animacy judgments

Children were asked four questions per item: three psychological (the ability to think, feel, and talk) and one biological (alive). Recall that a higher score reflects a higher level of anthropomorphism. Overall, 3-year-old children responded to the interview questions at chance (responding “Yes” about 50% of the time) across all four items: animals, artifacts, Dash, and Nao [all one sample *t* tests, *t*(88) < −1.23, *p* > 0.23]. In contrast, 5-year-old children attributed high levels of animacy to animals (*M* = 2.91, *SD* = 0.82, *t*(44) = 7.45, *p < *0.001, *d* = 1.11, 95% CI [0.73, 1.48]). The 5-year-old children also correctly did not anthropomorphize artifacts, performing below chance level (*M* = 0.47, *SD* = 0.73, *t*(44) = −14.17, *p < *0.001, *d* = −2.11, 95% CI [−2.64, −1.58]) and the non-humanoid robot Dash (*M* = 1.42, *SD* = 1.45, *t*(44) = −2.67, *p* = 0.01, *d* = −0.40, 95% CI [−0.70, −0.09]). The 5-year-old children were only at chance for Nao (*M* = 1.93, *SD* = 1.50, *t*(44) = −0.30, *p* = 0.77, *d* = −0.04, 95% CI [−0.34, 0.25]), suggesting they may be confused about the anthropomorphism of a humanoid looking robot (see Table 1 for a summary of main findings).

A repeated-measures analysis of variance test (ANOVA) was conducted to compare interview performance across the different items (Nao, Dash, animal, and artifact), with age as a between-subjects factor. A main effect of interview item [*F*(3) = 33.76, *p* < 0.001, ηp^2^ = 0.28] was found, with all items different except for the two robots. The main effect of age was trending on significance [*F*(1) = 3.17, *p* = 0.08, ηp^2^ = 0.04]. The interaction between performance and age was also significant [*F*(3) = 16.04, *p* < 0.001, ηp^2^ = 0.16]. The 5-year-old children performed significantly above the 3-year-olds only on the artifact trials, as shown by the *post hoc t*-tests [*t*(88) = 4.38, *p_holm_* < 0.001], see Figure 5.

##### Psychological properties

When considering just the three psychological questions (the ability to think, feel, and talk), and leaving out the biological question of alive, the 3-year-old children were still at chance for all items [all one-sample *t* tests, *t*(88) < −1.44, *p* > 0.16]. As with the total interview, 5-year-old children were above chance for attributing animacy to animals (*t*(44) = 4.25, *p* < 0.001, *d* = 0.63, 95% CI [0.31, 0.95]), below chance for both Dash (*t*(44) = −2.92, *p* = 0.005, *d* = −0.44, 95% CI [−0.74, −0.13]) and artifacts (*t*(44) = −12.98, *p* < 0.001, *d* = −1.94, 95% CI [−2.43, −1.43]), and at chance for Nao (*t*(44) = −0.06, *p* = 0.95, *d* = −0.009, 95% CI [−0.30, 0.95]).

##### Biological property

**Figure 6:**
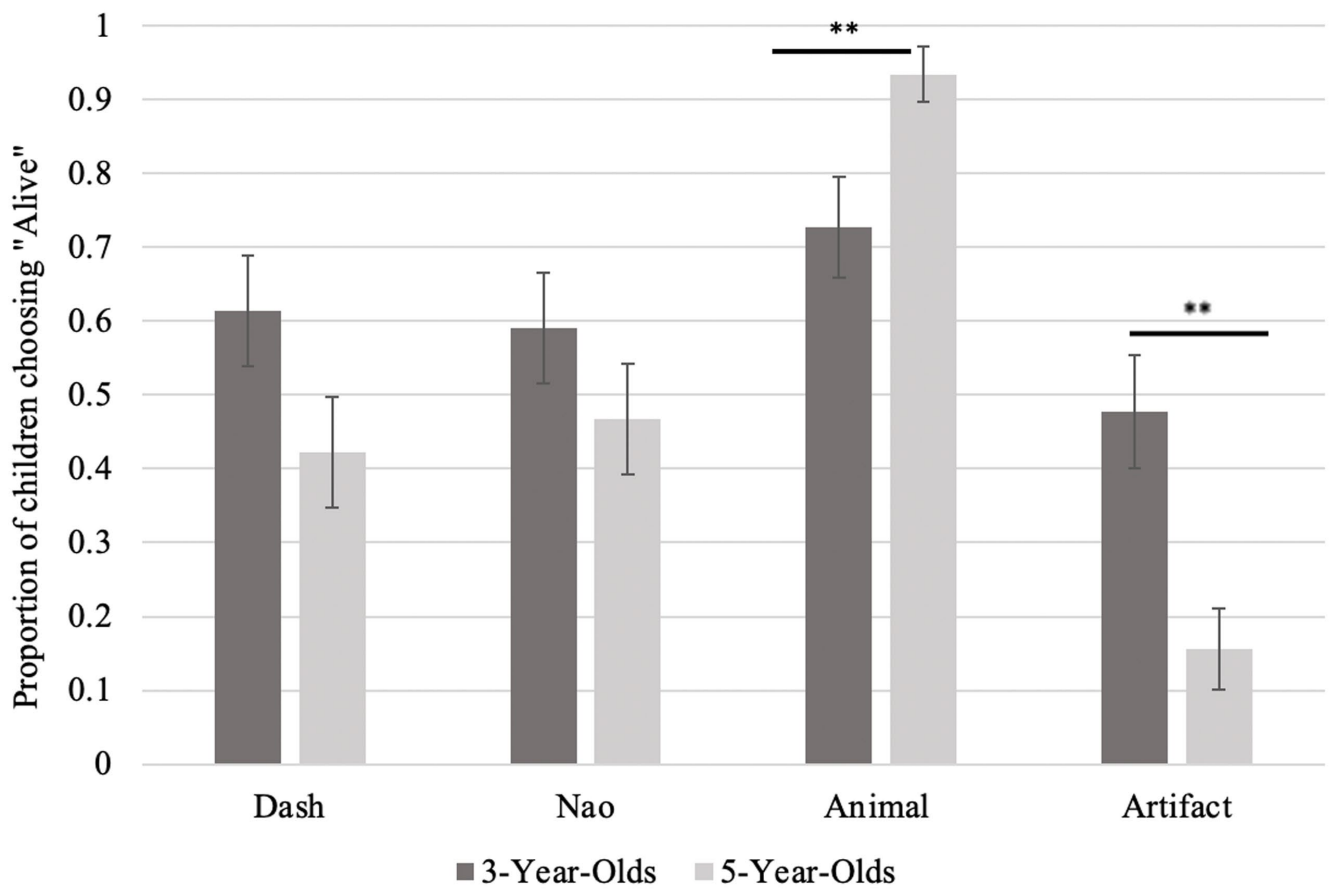
Proportion of children who rated each item as alive as a function of age. Percentage of children rating that item as alive. The ** (*p* < 0.01) indicates a significant difference between the two age group on that item.

For the one biological alive question, only the animal item was rated as alive by the 3-year-olds (*Prop* = 0.73, binomial test, *p* = 0.004, 95% CI [0.57, 0.85]), with Dash, Nao and the artifacts at chance (*Prop* = 0.61, *p* > 0.17). The 5-year-olds correctly answered that animals are alive (*Prop* = 0.93, *p* < 0.001, 95% CI [0.82, 0.99]) and artifacts are not alive (*Prop* = 0.84, *p* < 0.001, 95% CI [0.71, 0.94]). However, 5-year-olds were also at chance for both robots (*Prop* < 0.58, *p* > 0.37), which suggests they were confused about whether the robots were alive. An independent samples *t*-test, corrected for normality, found that 5-year-olds rated animals as significantly more alive than 3-year-olds (*Mann–Whitney* = 786.00, *p* = 0.01, *r_rb_* = −0.21, 95% CI [−0.42, 0.03]). The 5-year-olds also rated artifacts as significantly less alive than 3-year-olds (*Mann–Whitney* = 1308.50, *p* = 0.001, *r_rb_* = 0.32, 95% CI [0.09, 0.52]), and were trending to do the same for Dash (*Mann–Whitney* = 1179.50, *p* = 0.07, *r_rb_* = 0.19, 95% CI [−0.05, 0.41]). Performance was equal across the age groups for Nao (*Mann–Whitney* = 1113.00, *p* = 0.25, *r_rb_* = 0.12, 95% CI [−0.12, 0.35]), see Figure 6. See Table 1 for a summary of the main findings.

### Goal-directedness

All the children saw one robot exhibit goal-directed action and the other robot exhibit non-goal-directed action. A 2 × 2 repeated measure ANOVA comparing Naïve Biology performance for both Nao and Dash with either goal-directed or non-goal-directed behavior as a between subjects factor revealed no significant main effects of either robot type [Nao vs. Dash; *F*(1) = 1.12, *p* = 0.29, ηp^2^ = 0.01] or goal-directedness [*F*(1) = 0.07, *p* = 0.79, *ηp^2^* = 0.00]. The interaction between goal-directedness and robot type was significant [*F*(1) = 4.76, *p* = 0.03, *ηp^2^* = 0.05]. However, the *post-hoc* analyses revealed no significant *post-hoc* interaction *t*-tests [*t*(88) < −1.21, *p_holm_* > 0.15]. An independent samples *t*-test grouped by goal-directedness revealed a trending effect for Nao, with the children who saw Nao behave in a goal-directed manner performing slightly worse on the Naïve Biology task (Goal-Directed: *M* = 2.28, *SD* = 1.47, Non-Goal-Directed: *M* = 2.83, *SD* = 1.27, *t*(87) = 1.88, *p* = 0.06, *d* = 0.40, 95% CI [−0.02, 0.82]). No effect emerged for Dash (Goal-Directed: *M* = 2.94, *SD* = 1.18, Non-Goal-Directed: *M* = 2.54, *SD* = 1.39, *t*(87) = −1.47, *p* = 0.15, *d* = −0.31, 95% CI [−0.73, 0.11]).

An ANOVA comparing interview responses for all four questions for both Nao and Dash with either goal-directed or non-goal-directed behavior as a between-subjects factor found no significant main effects of either robot type [Nao vs. Dash; *F*(1) = 0.74 *p* = 0.39, ηp^2^ = 0.01] or goal-directedness [*F*(1) = 0.11, *p* = 0.74, ηp^2^ = 0.001]. No interaction between goal-directedness and robot type was found [*F*(1) = 1.79, *p* = 0.19, ηp^2^ = 0.02]. Therefore, the presence or absence of goal-directed behavior did not affect children’s animacy judgments of the robots.

### Inter-task analysis

Finally, we examined whether performance on one task was correlated with performance on the other task. Overall, the Animacy Interview and Naïve Biology task performance did not correlate for any of the items except artifacts: artifacts (*r*(87) = −0.27, *p* = 0.01, 95% CI [−0.45, −0.06]), Dash (*r*(87) = −0.18, *p* = 0.09, 95% CI [−0.38, 0.03]), Nao (*r*(87) = −0.02, *p* = 0.87, 95% CI [−0.23, 0.19]), and animals (*r*(87) = −0.19, *p* = 0.08, 95% CI [−0.38, 0.02]). It was predicted that children who correctly categorized the robot as not alive during the interview would score higher on the Naïve Biology task as well, assigning the robots a mechanical inside. The scores, however, were not significantly correlated (Nao: *r*(87) = 0.03, *p* = 0.81, 95% CI [−0.18, 0.23], Dash: *r*(87) = −0.09, *p* = 0.41, 95% CI [−0.29, 0.12]).

## Discussion

The current study explored how 3- and 5-year-old children conceptualized robots by administering both an Animacy Interview and a Naïve Biology task. The interview included questions that examined how children perceived the psychological (i.e., the ability to talk, think and feel) and biological (i.e., whether the robot is alive) aspects of robots. Interestingly, 3-year-olds performed at chance level for all the interview questions across the four items (artifact, animal, Nao, Dash). In contrast, 5-year-olds correctly attributed animacy to animals, knew artifacts, and the non-humanoid Dash robot was inanimate but were confused (performed at chance level) about the anthropomorphism of a humanoid-looking robot, Nao.

Focusing solely on the biological question, when asked whether robots, animals, or artifacts are alive, 3-year-olds in the present study performed at chance for both robots and the artifact, yet young children knew animals were alive. The 5-year-olds performed better, although they were also at chance for the robots, they knew that animals were alive, and artifacts were not. As both age groups knew animals were alive, this suggests that they understood the question. However, children of both age groups had difficulty assessing whether robots were alive. Upon examining the three interview questions that focused on the psychological domain, 3-year-olds were at chance for both robots, whereas 5-year-olds performed at chance only for the Nao robot. This suggests that young children do not yet have a solid understanding of robots, even when robots act and appear in ways that would suggest they are living (goal-directedness, autonomous movement, facial features, and appearance). Yet, 5-year-olds did not attribute animate psychological properties to Dash, the non-humanoid robot. We speculate that, for the 5-year-olds, who have more lived experiences, morphology played a larger role in their categorization and judgment of the robot than for 3-year-olds, who were just generally perplexed about both robots.

To avoid underestimating children’s competence in verbal interviews, we also administered a Naïve Biology task. The 3-year-olds attributed mechanical and biological internal parts to both robots (Nao and Dash) at equal rates regardless of the robot’s appearance (humanoid or non-humanoid). This suggests that, at the age of 3 years, children do not conceptualize robots clearly, unlike older children (aged 5 years and above) who perceive robots (both human looking and not) as artifacts but still anthropomorphize them, as adults do (Goldman et al., in press).

Overall, children performed better on the Naïve Biology task, which is a methodology better suited for testing young children than the interview questions, which have higher task demands. Using more applied tasks may be easier, especially for younger children. As 3-year-olds performed well on the Naïve Biology task for artifacts and animals, this suggests they understood the task, and their chance performance on the robot items (Nao, Dash) likely did not stem from the design of the task itself. We speculate that the 3-year-olds had difficulty categorizing robots as they often have characteristics of both living (e.g., morphology, autonomous movement, social behaviors) and non-living things (e.g., made of plastic or metal). Additionally, 3-year-olds may have limited experiences with and exposure to robots. In contrast, artifacts may be less ambiguous, as they only display characteristics of non-living things. Furthermore, young children may benefit from in-person interactions with robots as it may be easier to discern their features and characteristics compared to online testing. In contrast, older children in our sample (the 5-year-olds) knew that robots had mechanical insides and overwhelmingly assigned mechanical insides to both robots, regardless of the morphology of the robot. In fact, 5-year-olds excelled at the Naïve Biology task and correctly assigned mechanical insides to Nao, Dash, and artifacts, well also accurately assigning biological insides to animals.

A recent review by van Straten et al. (2019) found that altering a robot’s appearance or behavior yielded mixed results with children. van Straten et al. (2019) conclude that the impact of tailoring a robot to be similar to the child depends on whether the child notices the similarity between themself and the robot. In the present study, the morphology of the robots did not appear to affect how children in both age groups performed on the Naïve Biology task. However, for older children, morphology may have had more of an impact as children correctly did not anthropomorphize Dash, the non-humanoid-looking robot.

Interestingly, the presence or absence of goal-directed behavior in the robots did not change how children responded to either the Naïve Biology task or the Animacy Interview. We speculate that several reasons could explain such a null effect. First, perhaps the goal-directed behavior (i.e., the robot moving toward or away from a ball) was unclear to children this age. However, autonomous movement is a powerful cue of animacy, and even infants attribute goals and beliefs to self-propelled, goal-directed artifacts like a toy crane (Shimizu and Johnson, 2004; Luo, 2011; Burnside et al., 2020). Additionally, both robots tilted their heads and directed their eyes towards either the toy ball or towards the other side of the screen. Therefore, given that the robots displayed the social cue of eye gaze, it is likely that children perceived the robot as a social agent. Another possibility is that children saw a single video for each robot, and each video was quite short (approximately 8 s in length). Research has suggested that infants and young children may benefit from multiple examples of a behavior in order to conclude it is a goal-directed action (e.g., Maguire et al., 2008; Snape and Krott, 2018). Future work could aim to make the goal-directed actions of the robot clearer by choosing a less ambiguous action (e.g., reaching for an object or verbally expressing a goal). Importantly, research has revealed that robots do have greater influence when physically present compared to when they are shown *via* video (Bainbridge et al., 2011; Li, 2015). For example, Bainbridge et al. (2011) found that participants cooperated with a robot that was physically present or displayed *via* a video at equal rates but were more likely to fulfill an unusual request if a robot was physically present. Thus, the goal-directed behavior presented in videos may have been more challenging for children to understand than if the behavior had been demonstrated live in real-time. Future work that aims to examine the impact of goal-directed behavior on children’s conceptualizations of robots should be conducted in person. Lastly, goal-directedness may be less of an important agency characteristic compared to others, such as autonomous movement or speech.

The present work offers unique contributions to the existing body of research, including a novel approach to examining children’s conceptualization of robots. Firstly, the study included two robots that differed significantly in their morphology. As much of the prior work examined children’s conceptualizations of a single robot (Breazeal et al., 2016; Chernyak and Gary, 2016; Peca et al., 2016; Brink and Wellman, 2020; Manzi et al., 2020) using two robots with different morphologies allowed us to directly examine what role, if any, morphology plays in young children’s conceptualization of robots. Secondly, testing children’s conceptualization of robots with a Naïve Biology task that included robots in addition to animals and artifacts has not, to our knowledge, been done before. Thirdly, the current study used two tasks to provide a more complete understanding of children’s perceptions of robots. Specifically, the Naïve Biology task provides insight into children’s biological conceptions of robots, while the Animacy Interview offers children’s understanding of both a robot’s psychological abilities and biological status. Finally, the present work pits the morphology of the robots against the animacy characteristic of goal-directedness. As robots often exhibit a variety of animate characteristics, we opted to examine whether the presence or absence of goal-directed behavior would impact children’s conceptualizations of these robots.

In sum, the present findings suggest that very young children may be confused about the nature of robots in general and that this confusion does not mainly stem from the robot’s morphology. There are a number of opportunities to build upon the present work. An important direction for future research will be to use other experimental tasks in addition to interviews. It would also be useful to include an adult group to confirm how they would perform on a more applied task like the Naïve Biology task. Additionally, future research could extend upon the present study by asking children to provide justifications for their responses to the interview questions. This would provide a better understanding of why children are or are not attributing animate properties to the robots. Another interesting extension to the present work would be a cross-cultural study examining how children’s varying experience with robots could affect their conceptualization of them. Finally, in addition to goal-directedness, other cues (e.g., speech, eye gaze, contingency, gestures) could be examined to determine how the presence or absence of such cues impacts young children’s perceptions of robots.

As children in the present study always saw the video of the robot moving first, the autonomous movement the robots demonstrated may have impacted children’s conceptualization of the robots. To test this, future work could use static images of the robots in place of the videos. As much of the existing work has relied primarily on interviews to assess children’s conceptualizations of robots (Jipson and Gelman, 2007; Beran et al., 2011; Chernyak and Gary, 2016; Manzi et al., 2020), the present work takes an important step in building a more complete understanding of how children perceive social robots. Using a range of tasks (i.e., Naïve Biology and Animacy Interview), we assessed young children’s perceptions of robots that varied in their morphology. This work has provided initial insights into multiple domains (e.g., psychological, biological) of children’s conceptualizations of social robots and shows how young children’s conceptualizations change over time.

## Data availability statement

The raw data supporting the conclusions of this article will be made available by the authors, without undue reservation.

## Ethics statement

The studies involving human participants were reviewed and approved by Concordia University Human Research Ethics Committee. Written informed consent to participate in this study was provided by the participants’ legal guardian/next of kin.

## Author contributions

EG, A-EB, and DP-D conceived and designed the experiment and wrote and revised the manuscript. EG collected data and granted the use of the Wonder Workshop robot Dash. A-EB and EG carried out statistical analyses. DP-D granted the use of the SoftBank Robotics Nao humanoid robot. All authors contributed to the article and approved the submitted version.

## Funding

This work was supported by an Insight Grant provided by the Social Sciences and Humanities Research Council of Canada (#435-2022-0805) awarded to DP-D.

## Conflict of interest

The authors declare that the research was conducted in the absence of any commercial or financial relationships that could be construed as a potential conflict of interest.

## Publisher’s note

All claims expressed in this article are solely those of the authors and do not necessarily represent those of their affiliated organizations, or those of the publisher, the editors and the reviewers. Any product that may be evaluated in this article, or claim that may be made by its manufacturer, is not guaranteed or endorsed by the publisher.
